# IgA anti-β2-glycoprotein I as an independent risk factor in acute venous thromboembolism

**DOI:** 10.3389/fimmu.2026.1818399

**Published:** 2026-07-20

**Authors:** Raquel Diaz-Simon, Antonio Lalueza, Manuel Serrano, Daniel Pleguezuelo, Oscar Cabrera-Marante, David Lora, Carmen Diaz-Pedroche, Covadonga Gomez-Cuervo, Asunción Perez-Jaicoste, Sara Garcinuño, Laura Naranjo, Elena Martinez-Chamorro, Yolanda Revilla-Ostolaza, Estela Paz-Artal, Carlos Lumbreras-Bermejo, Antonio Serrano

**Affiliations:** 1 Internal Medicine Department, Hospital Universitario 12 de Octubre, Madrid, Spain; 2 Faculty of Medicine, Universidad Complutense de Madrid, Madrid, Spain; 3 Instituto de Investigación Hospital 12 de Octubre (imas12), Hospital Universitario 12 de Octubre, Madrid, Spain; 4 Immunology Department, Hospital Universitario 12 de Octubre, Madrid, Spain; 5 Facultad HM de Ciencias de la Salud, Universidad Camilo José Cela, Villanueva de la Cañada, Madrid, Spain; 6 Instituto de Investigación Sanitaria HM Hospitales, Madrid, Spain; 7 Radiology Department, Hospital Universitario 12 de Octubre, Madrid, Spain

**Keywords:** anti beta 2 glycoprotein I, antiphospholipid antibodies, chronic thromboembolic pulmonary hypertension, IgA, venous thromboembolism

## Abstract

**Background:**

Venous thromboembolism (VTE) is the third most common cause of cardiovascular death. Primary Antiphospholipid Syndrome (P-APS) is a chronic systemic autoimmune disorder characterized by thrombotic events and/or obstetric complications in patients carrying antiphospholipid antibodies (aPL) without autoimmune pathology. The prevalence of primary antiphospholipid syndrome (P-APS) in patients with VTE is approximately 9%. However, few studies have evaluated non-criteria antiphospholipid antibodies in the acute phase of VTE.

**Objective:**

To assess the role of criteria and non-criteria antiphospholipid antibodies in patients with acute VTE.

**Methods:**

A cohort of 181 patients with VTE was followed for 2 years. An age-matched control group of 181 healthy individuals was included for comparison.

**Results:**

Criteria aPL were detected in 8.8% of patients, whereas non-criteria aPL were present in 23.8%. Criteria aPL were independently associated with overall VTE (OR 7.09, p=0.021) and unprovoked VTE (OR 4.61, p=0.021). IgA anti-Beta2 Glycoprotein-1 antibodies (aB2GP1), detected in 16% of patients, were independently associated with VTE (OR 4.31, p=0.014) and unprovoked VTE (OR 3.78, p=0.006). Furthermore, IgA aB2GP1 positivity was also associated with more severe clinical presentation, defined by a Pulmonary Embolism Severity Index scale ≥3 (OR 3.13; p=0.048), and with the development of chronic thromboembolic pulmonary hypertension (OR 5.7; p=0.008).

**Conclusion:**

IgA aB2GP1 antibodies are independently associated with VTE, particularly unprovoked VTE, and with both severe pulmonary embolism, and subsequent chronic thromboembolic pulmonary hypertension.

## Introduction

Cardiovascular diseases (CVDs) are the leading cause of global mortality, accounting for 17.3 million deaths in 2008 and an estimated 23.6 million by 2030 (1). Venous thromboembolism (VTE), encompassing deep vein thrombosis (DVT) and pulmonary embolism (PE), represents the third most common cause of vascular death worldwide (2). Its incidence continues to increase, partly due to population aging. Adjusted for age, body mass index (BMI), ethnicity, cancer and antithrombotic therapy, VTE incidence ranges from 0.87–1.82 per 1,000 person-years in high-income countries and 0.45–0.95 per 1,000 person-years in low-income countries (3, 4). Venous thrombosis results from the interplay of genetic, environmental and behavioural risk factors, with malignancy and major surgery—particularly orthopaedic and neurosurgical procedures—being among the most significant contributors (5).

Antiphospholipid syndrome (APS) is a multisystem autoimmune disorder characterized by venous, arterial or small-vessel thrombosis and/or pregnancy morbidity in the presence of antiphospholipid antibodies (aPL) (6, 10). Although no formal diagnostic criteria exist, the classification criteria proposed at the Eleventh International Congress on Antiphospholipid Antibodies (Sydney, 2004) are widely used as a diagnostic guide (8). These require clinical evidence of thrombosis or obstetric morbidity, plus laboratory confirmation of lupus anticoagulant (LA), anticardiolipin antibodies (aCL IgG or IgM), or anti-β2-glycoprotein I antibodies (aβ2GPI IgG or IgM) on two occasions at least 12 weeks apart (8). Updated classification criteria have recently broadened the clinical spectrum and reduced the diagnostic weight of IgM isotypes (9).

Three forms of APS are recognized (10): (1) APS associated with a systemic autoimmune disease (SAD-APS), mainly systemic lupus erythematosus (SLE) (11); (2) primary APS (P-APS), in which there is no association with any autoimmune disease; and (3) catastrophic APS, a rapidly progressive form, with thrombosis in multiple territories, that is associated with high mortality (11). P-APS is the most common form, with 9 P-APS cases for each SAD-APS case. However, most APS studies focus on SAD-APS patients, which may lead to under-recognition of P-APS during VTE evaluation.

Some patients who suffer from unexplained thrombosis are negative for criteria aPL. To describe these patients, Hughes and Khamashta coined the concept of “seronegative APS” (6). This may result from insufficient test sensitivity, low titres of antibodies, or antibodies directed against antigens other than cardiolipin or aβ2GPI (7, 20). These antibodies are known as non-criteria aPL (7, 20). Although many non-criteria aPL have been described, those with greatest clinical relevance are anti-phosphatidylserine/prothrombin (aPS/PT) and IgA anti-β2GPI (IgA aβ2GPI) (20, 21). The potential clinical and pathogenic relevance of IgA aβ2GPI remains controversial, with heterogeneous evidence regarding its association with thrombotic events (19, 20).

APS has been reported in approximately 9% of individuals with a first VTE event (22), and aPL positivity is associated with an increased risk of recurrence (32, 33). However, important knowledge gaps remain. Few studies have evaluated: (i) the prevalence of aPL in the acute phase of VTE; (ii) the impact of criteria and non-criteria aPL on morbidity and mortality; and (iii) the prognostic value of non-criteria aPL, particularly IgA aβ2GPI and aPS/PT.

## Materials and methods

### Study design

This is an observational, prospective, and single-centre study.

Main goal. Cases and controls study. To demonstrate that patients who suffer VTE have higher aPL antibodies prevalence compared to the general population.

Secondary objective. Case tracking. To analyse the association between the presence of both criteria and non-criteria aPL antibodies and their impact on morbidity and mortality caused to VTE.

### Patients and controls

A total sample of 362 subjects was analysed, comprising 181 cases and 181 controls. The sample size was calculated based on an expected event rate of approximately 12% among cases (23) versus 4% among controls (24), providing a statistical power of 80% and an alpha level of 0.05.

The study group included 181 adult patients with acute VTE who were consecutively recruited between January 2018 and June 2019 upon admission to the 12 de Octubre University Hospital. Most of the patients (93%) were of Caucasian ethnicity.

The control group comprised of 181 individuals from the same geographical area as the patient cohort, with a similar distribution in terms of ethnic background and age. Controls were recruited from two populations: blood donors under the age of 65 and elderly individuals attending the pre-anaesthetic ophthalmology consultations for refractive disorders. None of the control subjects had a known diagnosis of vascular disease, autoimmune disorder or malignancy.

Inclusion criteria: Adult patients (≥18 years) admitted to the hospital for DVT and/or PE, confirmed by venous Doppler ultrasound and/or computed tomography pulmonary angiography who provided informed consent.

Exclusion criteria: patients with VTE and concurrent active malignancy or a diagnosis of systemic autoimmune disease were excluded from the study.

Patients with active malignancy were excluded due to the confounding effect of cancer-associated antiphospholipid antibody positivity, whose pathogenic role in VTE remains uncertain, which would have compromised the reliability of thrombophilia-related analyses.

### Ethical issues

The study was approved by the Hospital 12 de Octubre Clinical Research Ethical Committee (references CEIC-14/354 and CEIM-18/182). Both patients and controls provided informed consent for the collection of these samples and their subsequent analysis.

### Definitions

- Acute venous thromboembolism (VTE): condition characterized by thrombus formation in the deep venous system (deep vein thrombosis, DVT), which may propagate or embolize to the pulmonary arteries, resulting in pulmonary embolism (PE) (41).
- Pulmonary embolism severity: assessed using the Pulmonary Embolism Severity Index (PESI), a validated prognostic model incorporating clinical variables including age, sex, comorbidities, vital signs, mental status, and oxygen saturation (29).
- Thromboembolic recurrence: defined as a new objectively confirmed venous thromboembolic event (32).
- Respiratory failure: defined as a PaO_2_/FiO_2_ (PaFi) ratio <300, where PaO_2_ represents arterial oxygen partial pressure and FiO_2_ the fraction of inspired oxygen (25).
- Obesity: defined as body mass index (BMI) ≥30 kg/m² according to World Health Organization criteria.
- Dyslipidemia: defined as total cholesterol ≥200 mg/dL, LDL cholesterol ≥130 mg/dL, HDL cholesterol ≤40 mg/dL, or triglycerides ≥150 mg/dL, according to ESC/EAS guidelines (26).
- Hypertension: defined as systolic blood pressure ≥130 mmHg and/or diastolic blood pressure ≥80 mmHg on at least two measurements or current antihypertensive treatment, according to ESC/ESH guidelines (27).
- Post-thrombotic syndrome: defined as signs and symptoms of chronic venous insufficiency following DVT, assessed using the Villalta scale (28).
- Chronic thromboembolic disease: defined as persistence of thrombotic material in the pulmonary vascular bed beyond 3–6 months after acute PE (35).
- Chronic thromboembolic pulmonary hypertension (CTEPH): defined as precapillary pulmonary hypertension (mean pulmonary arterial pressure >20 mmHg) confirmed by right heart catheterization, with persistent perfusion defects after at least 3 months of effective anticoagulation (35, 36).
- Haemorrhagic complications: defined as clinically relevant non-major bleeding or major bleeding according to ISTH criteria (40). Major bleeding was defined as fatal bleeding, symptomatic bleeding in a critical area, or a drop in haemoglobin ≥2 g/dL or transfusion of ≥2 units of red blood cells. Clinically relevant bleeding was defined as bleeding requiring medical intervention, hospitalization, or increased level of care.
- Major risk factors for thrombosis: included transient or persistent factors such as hospitalization, immobility, trauma, pregnancy/puerperium, hormone therapy, or active cancer, as well as inherited or acquired thrombophilia (5, 41).
- Provoked VTE: defined as VTE occurring in the presence of at least one major risk factor for thrombosis (41).
- Unprovoked VTE: defined as VTE occurring in the absence of identifiable major risk factors (41).
- Criteria antiphospholipid antibodies (criteria aPL): defined as lupus anticoagulant (LA), anticardiolipin antibodies (aCL IgG/IgM), and anti-β2-glycoprotein I antibodies (aβ2GPI IgG/IgM), according to Sydney classification criteria (8, 9).
- Triple aPL positivity: defined as the simultaneous presence of LA, aCL, and aβ2GPI antibodies (33).

### Laboratory determinations

The samples to detect the presence of criteria and non-criteria aPL antibodies were extracted in the first 24 hours after admission at the hospital and were sent to the Autoimmunity Laboratory of the Immunology Department at 12 de Octubre University Hospital.

The aCL and aB2GP1 antibodies (IgG and IgM isotypes) were quantified using the BioPLex 2200 multiplex immunoassay system APLS (Bio-Rad, Hercules CA, USA). Antibody levels greater than 18 U/ml were considered positive (99th percentile of a healthy population, N = 270). The BioPlex 2200 system uses ALBIA (Addressable Laser Bead Immunoassay) technology, which employs a logarithmic detection scale and has a narrower dynamic range compared with conventional ELISA. As a consequence, absolute antibody values obtained with this platform are systematically lower than those reported with ELISA-based assays. In a validation study of 1,018 individuals (252 patients and 766 healthy controls), the median and modal antibody levels in the healthy population were below 1 U/ml, confirming that the 99th-percentile threshold of 18 U/ml is appropriate for this technology and that titre-based stratification into low, medium and high categories does not provide additional clinical utility with this platform (46).

LA was evaluated using coagulation assays according to the recommendations of the ISHT (32, 33) using the HemosIL dRVVT Screen, HemosIL dRVVT Confirm and HemosIL Silica Clotting Time assays (Instrumentation Laboratory SpA, Milano, Italy).

The IgA aB2GP1 antibodies were quantified by enzyme-linked immunosorbent assay (ELISA) using the QUANTA Lite B2 GPI IgA (INOVA Diagnostics Inc., San Diego, CA, USA). The aPS/PT antibodies (IgG and IgM isotypes) were assessed using QUANTA Lite aPS/PT (INOVA DIAGNOSTICS, San Diego, CA, USA). The cut-off values defined as positive were >20 U/ml for IgA aB2GP1, >30 U/ml for IgG aPS/PT and >40 U/ml for IgM aPS/PT antibodies. The cut-off points were set using the 99th percentile of a representative group of the healthy population (N = 718) which had the same ethnic origin and area of residence as the patients. These thresholds were selected according to current international recommendations for antiphospholipid antibody testing and manufacturer validation studies.

Of the 181 patients tested, 51 (28.2%) were positive for aPL antibodies. Among them, a second confirmatory determination was performed in 35 patients (69%) at least 12 weeks after the initial assessment, in accordance with classification criteria.

### Databases and statistical methods

Demographic and clinical characteristics were described by absolute frequency and percentages for qualitative variables, and as medians and interquartile ranges (IQR) for quantitative variables. The association between cases and controls, baseline characteristics and aPL antibodies were examined using Pearson’s χ2 test or Fisher’s exact test where appropriate. The comparison between cases and controls in quantitative variables was carried out using the non-parametric Mann Whitney U test.

To establish the association between the case-control variable and the independent variables we performed binary logistic regression in a univariate manner. The association was quantified using the odds ratio (OR), along with its 95% confidence interval and its statistical significance. Multivariable analyses were conducted using a logistic regression model. The results were accompanied by the area under the diagnostic performance curve or ROC curve (Receiver Operating Characteristic) along with its 95% confidence interval. To analyze mortality univariate binary logistic regression was used, and a multivariate analysis was performed on significant variables.

Univariate binary logistic regression was performed to analyse recurrence, bleeding, and complication events for a 24-month period after hospitalization. The final multivariate logistic regression models were built considering both those risk factors with a p-value less than 0.1 in the univariate analysis or those that had some interest or relevance within the study. Cox regression was not used due to the short follow-up (less than 5 years). A two-sided alpha error of 5% was used as the significance level for all contrasts. The data analysis was conducted using SPSS 28.0.2.2 statistical software (IBM SPSS Statistics 20; Chicago, IL, USA) and Medcalc 20.1 (Medcalc Software, Ostend, Belgium).

## Results

### Baseline characteristics

Among the 181 patients with VTE, women accounted for 57%, with no significant difference compared with men (p=0.832). The median age at the acute VTE event was 76 years (IQR 55–85). Women were significantly older than men (median 81 years, IQR 62–86 vs. 65 years, IQR 49–81; p=0.009). Traditional cardiovascular risk factors—including hypertension, dyslipidemia, obesity and smoking—as well as coronary artery disease, were significantly more prevalent in VTE patients than in controls.

**Figure 1:**
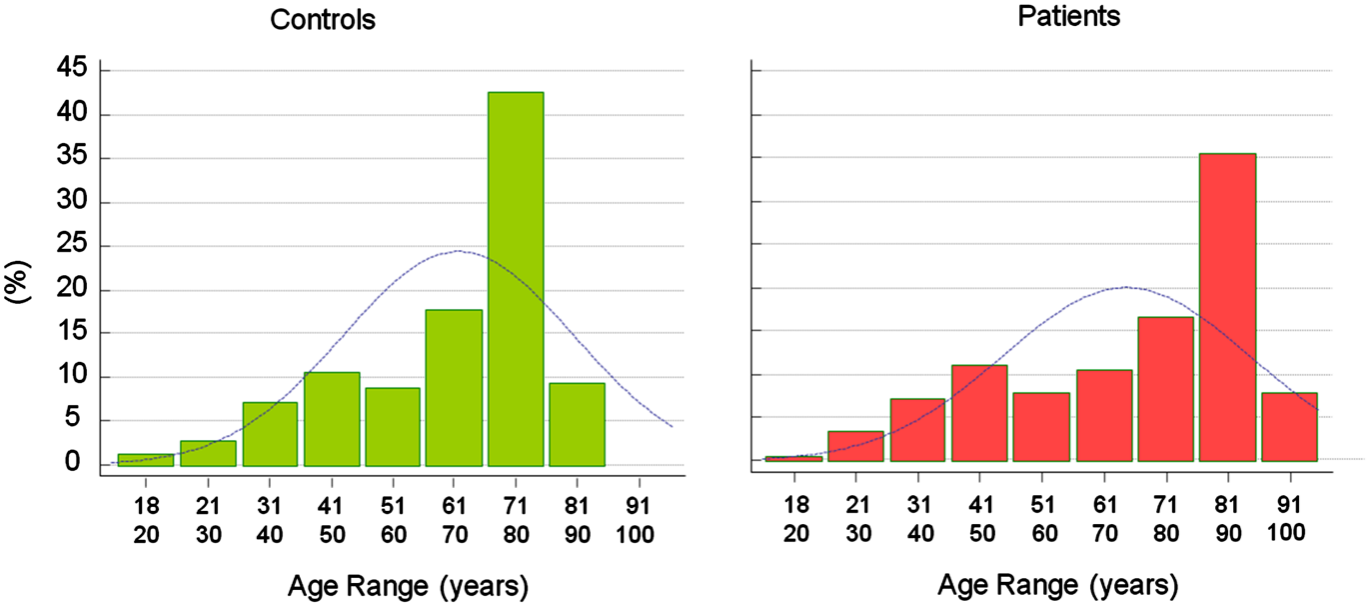
Histograms showing age distribution in patients and controls.

Although cases and controls were matched for age, VTE patients were slightly older overall (median 70 years, IQR 55–76; p=0.012), suggesting incomplete matching and potential residual confounding. Notably, the proportion of subjects aged >65 years did not differ significantly between groups (**Figure 1**). Clinical characteristics of patients and controls are shown in **Table 1**.

**Table 1.** Baseline and demographic characteristics of the patients and controls.

Parameter	Patients (n=181)	Controls (n=181)	OR (CI 95%)	p (value)
Clinical characteristics				
Age over 65 years	116 (52%)	108 (48%)	1.21 (0.79-1.84)	0.387
Gender (women)	101 (56%)	103 (57%)	1.04 (0.69-1.59)	0.832
Hypertension	105 (58%)	67 (22%)	**2.35 (1.54-3.58)**	**<0.001**
Diabetes	27 (15%)	27 (15%)	1 (0.56-1.78)	1
Dyslipidemia	65 (36%)	47 (26%)	**1.60 (1.02-2.51)**	**0.041**
Smoking	56 (31%)	25 (14%)	**2.79 (1.65-4.73)**	**<0.001**
Obesity	70 (39%)	20 (11%)	**7.6 (4.31-13.43)**	**<0.001**
Artery coronary disease	16 (9%)	2 (1.1%)	**8.73 (1.98-38.6)**	**0.001**
Cerebrovascular Disease	14 (8%)	0 (0%)	–	0.999
Antiphospholipid antibodies positivity				
Criteria aPL	16 (8.8%)	4 (2.2%)	**4.30 (1.40-13.10)**	**0.010**
aCL (IgG or IgM)	13 (7.2%)	4 (2.2%)	**3.42 (1.09-10.7)**	**0.043**
IgG aCL	7 (3.9%)	0 (0%)	15.60 (0.87-260)	0.061
IgM aCL	8 (4.4%)	4 (2.2%)	2.04 (0.605-6.920)	0.240
aB2GP1 (IgG or IgM)	12 (6.6%)	3 (1.7%)	**4.21 (1.17-15.19)**	**0.032**
IgG aB2GP1	5 (2.8%)	0 (0%)	NE	0.999
IgM aB2GP1	9 (5%)	3 (1.7%)	3.10 (0.827-11.661)	0.078
Non-criteria aPL	43 (23.8%)	20 (11%)	**2.51 (1.41-4.47)**	**0.001**
IgA aB2GP1	29 (16%)	9 (5%)	**3.65 (1.67-7.95)**	**0.001**
Any aPS/PT (IgG or IgM)	21 (11.6%)	12 (6.6%)	1.84 (0.881-3.880)	0.103
IgG aPS/PT	9 (5%)	5 (2.8%)	1.84 (0.605-5.607)	0.276
IgM aPS/PT	16 (8.8%)	7 (3.9%)	2.41 (0.97-6)	0.053
Any aPL	50 (27.6%)	24 (13.3%)	**2.50 (1.46-4.28)**	**<0.001**
Lupus anticoagulant*	13 (7.7%)	10 (6.3%)	1.27 (0.54-3.00)	0.577

NE: Not evaluable, all controls are negative, much larger samples would be necessary to calculate it. Abbreviations: aPL, antiphospholipid antibodies; aCL, anticardiolipin; aB2gp1, Anti-Beta2 glycoprotein 1; aPS/PT, anti-phosphatidylserine-prothrombin.

* Lupus anticoagulant was evaluated in 168 patients and 162 controls.

Significant results are marked in bold.

### Prevalence of aPL

A total of 51 VTE patients (28.2%) tested positive for at least one aPL. The prevalence of criteria aPL was 8.8%. Individually, IgG aCL was present in 3.9%, IgM aCL in 4.4%, IgG aB2GPI in 2.8% and IgM aB2GPI in 5.0%. No significant differences were observed between patients and controls for these antibodies (**Table 1**). Lupus anticoagulant (LA) was assessed in 168 patients, with 13 (7.2%) testing positive, a prevalence similar to controls (6.3%, p=0.577). Most positive antibodies were detected at low-to-moderate titres; however, due to the limited number of antibody-positive patients, stratified analyses according to antibody titre categories were not performed.

**Figure 2:**
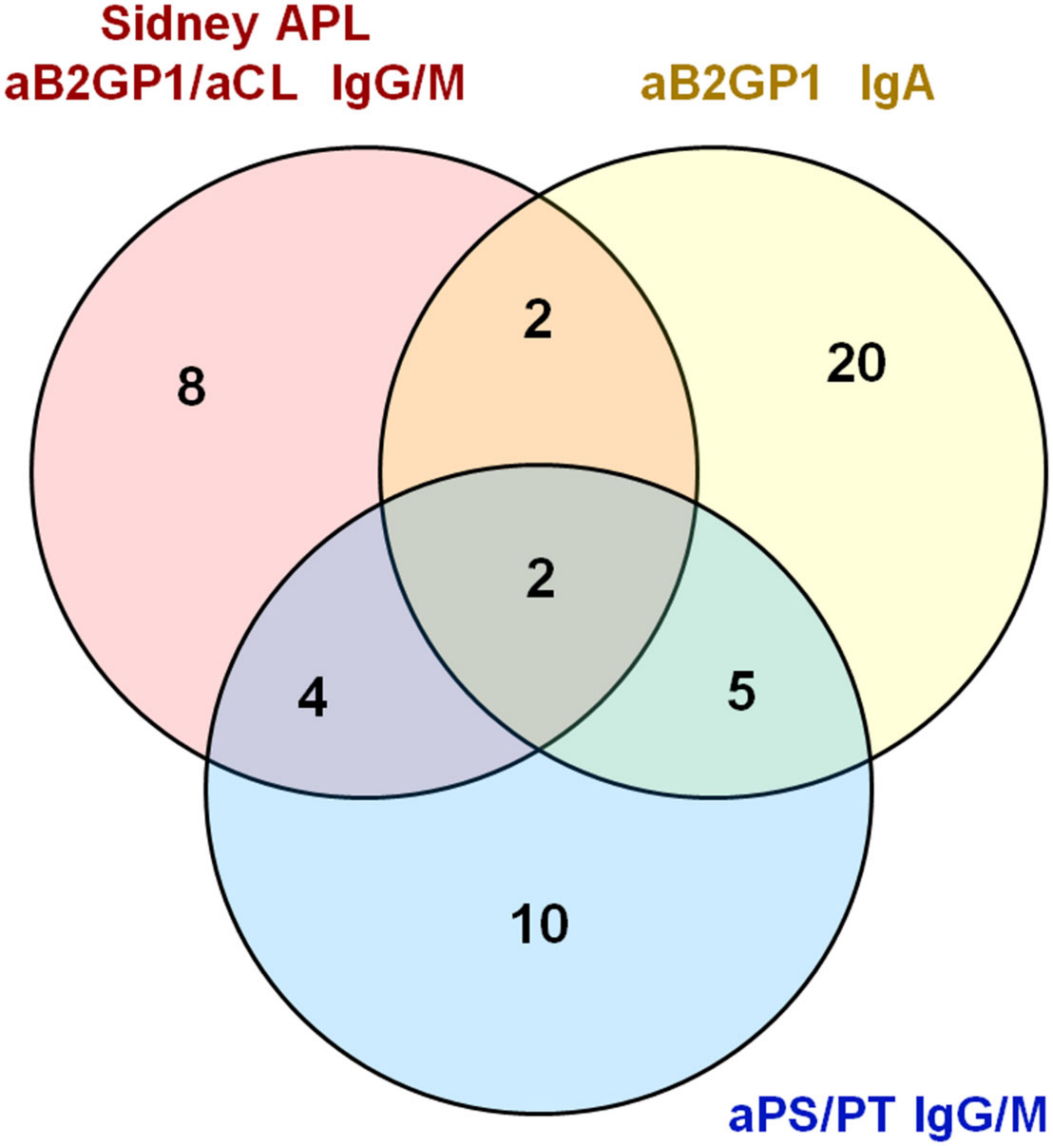
Venn diagram showing distribution of positivises for the different aPL in patients with VTE.

Autoantibody titter distributions and cut-off points are shown in **Supplementary Figure 2**. A Venn diagram illustrating antibody overlap among aPL-positive patients is provided in **Figure 2**.

**Figure 3:**
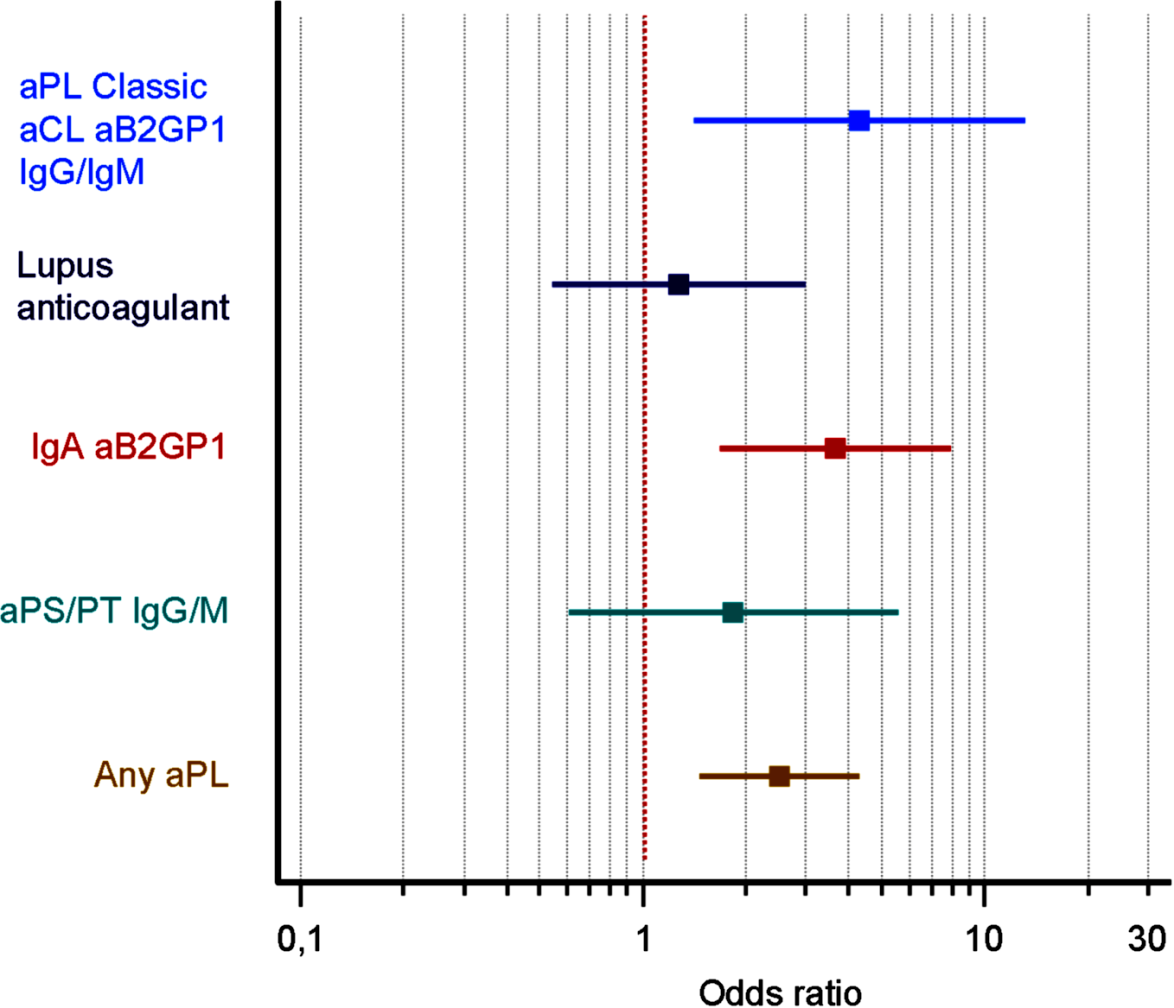
Odds ratio plot of antiphospholipid antibodies association with VTE.

Compared with controls, VTE patients had a significantly higher prevalence of criteria aPL (OR 4.3, 95% CI 1.40–13.10), non-criteria antibodies (OR 2.51, 95% CI 1.41–4.47) and IgA aβ2GPI (OR 3.6, 95% CI 1.67–7.95) (**Figure 3**; **Table 1**).

### Confirmation of aPL positivity

The study reassessed aPL positivity in 35 of the 51 aPL-positive patients (69%) at least 12 weeks after the initial determination, in accordance with current classification recommendations. Persistent positivity was confirmed in 89% of retested individuals. Positivity for criteria aPL was confirmed in all reassessed patients, while IgA aβ2GPI antibodies remained positive in 95% of cases (see **Supplementary Table 1**).

### Risk factors associated with VTE

Variables associated with VTE at a significance level of p value < 0.1 -hypertension, coronary artery disease, obesity, dyslipidemia, smoking, criteria aPL, IgA aB2GP1 antibodies and IgM aPS/PT; antibodies- (**Table 1**), were included in a multivariate logistic-regression analysis (**Table 2**).

**Table 2:** Multivariate analysis of the risk factors associated with VTE.

Variable	OR (95% CI)		P value
Hypertension	1.33	0.74-2.37	0.341
Artery coronary disease	8.11	**1.55-42.52**	**0.013**
Obesity	7.49	**3.95-4.19**	**<0.001**
Dyslipidemia	1.32	0.73-2.41	0.362
Smoking	3.78	**1.97-7.27**	**<0.001**
Criteria aPL	4.37	**1.15-6.62**	**0.031**
aB2GP1 IgA	3.37	**1.33-8.59**	**0.011**
aPS/PT IgG/M	2.98	**0.95-9.32**	**0.06**
**Area under ROC curve**	0.809	(0.762- 0.851)	

aPL, antiphospholipid antibodies; aCL, anticardiolipin; aB2GP1,

Anti-Beta2 glycoprotein 1; aPS/PT, anti-phosphatidylserine-prothrombin.

Significant results are marked in bold.

Five factors were independently associated with VTE: coronary artery disease (8.11, 95% CI:1.55-42.52); obesity (7.49, 95% CI:3.95-4.19); smoking (3.78, 95% CI:1.97-7.27); criteria aPL positivity (4.37, 95% CI:1.15-6.62), and IgA aB2GP1 positivity (3.37, 95% CI:1.33-8.59).

In a secondary multivariable analysis, in which age was modelled as a continuous variable (in years), this relationship was not altered, as age remained non-significant (OR 1.01; 95% CI 0.99-1.03). Conversely, all other variables previously identified as independent predictors retained their statistical significance and independent effects (**Supplementary Table 2**).

#### Unprovoked VTE

A total 114 patients (63%) experienced an unprovoked VTE. When compared with controls (refer to **Supplementary Table 2**), five variables were significantly associated with unprovoked VTE: hypertension (OR 2.51 (95% CI 1.55-4.06), dyslipidemia (OR 1.66, 95% CI 1.00-2.75; p=0. 048), obesity (OR: 8.59, 95% CI 4.62-15.99); smoking (OR 3.12, 95% CI 1.75-5.54); positivity for criteria aPL (OR 4.72, 95% CI 1.46-15.22; p<0.001) and positivity for IgA aB2GP 1 (OR 4.83, 95% CI 2.14-15.22; p<0.001).

A multivariate logistic regression analysis was conducted including the six risk factors with a p-value < 0.1 (the five variables and age >65 years, p=0.095; see **Table 3**). The resulting model demonstrated good discriminatory ability (ROC curve: 0.831). Four independent risk factors were identified: obesity (OR 9.55, 95% CI 4.57-9.98), smoking (OR 6.03, 95% CI 2.86-12.72), criteria aPL positivity (OR 5.57, 95% CI 1.37-22.74) and IgA aB2GP1 positivity (OR 4.15, 95% CI 1.5-11.51).

**Table 3:** Non-Provoked VTE Multivariate analysis.

Variable	OR (95% CI)		P value
Age over 65	1.29	0.61-2.73	0.508
Hypertension	1.34	0.65-2.76	0.434
Dyslipidemia	1.16	0.56-2.39	0.693
Obesity	9.55	**4,57-19,98**	**<0.001**
Smoking	6.03	**2,86-12,72**	**<0.001**
Criteria aPL	5.57	**1,37-22,74**	**0.017**
aB2GP1 IgA	4.15	**1,5-11,51**	**0.006**
Area under ROC curve	0.831	(0.781-0.874)	

aPL, antiphospholipid antibodies; aB2gp1, Anti-Beta2 glycoprotein 1.

Significant results are marked in bold.

In a subsequent multivariable analysis, in which age was modelled as a continuous variable (in years), a statistically significant association was demonstrated, albeit of a modest magnitude (OR 1.03; 95% CI: 1.01-1.06). The remaining variables in the model remained significant and independent (**Supplementary Table 4**).

### Two-year follow-up

#### Mortality

Thirty-two patients died during the follow-up period. The all-cause mortality rate was 19% with an annual mortality rate of 9.5%. Median survival was 404 days (IQR 52-624). Seven patients (21%) died during the acute phase of VTE, and the remaining deaths occurred during follow-up. Patients who died were older (median 86 years, IQR 76-90) compared to survivors (median 72 years, IQR 49-83; p=0.006).

Factors significantly associated with mortality included age over 65 years (OR 3.96, 95% CI 1.49-10.48), hypertension (OR 2.79, 95% CI 1.18-6.58), neurological disease (OR 4.7,95% CI 2.15-10.31), cerebrovascular disease (OR 7.23, 95% CI 2.31-22.56); and more severe VTE forms at presentation (OR 4.54 (95% CI 1.28-16.05) (**Supplementary Table 3**).

In multivariate analysis, the only independently variable associated with mortality was cerebrovascular disease (OR:4.54, 95%CI 1.06-19.43).

#### Thrombotic recurrences

Sixteen patients (9.2%) experienced recurrent thrombosis. The median time to recurrence was 17 months (IQR: 10-18), with an annual recurrence rate of 4.6%. Among those who recurred, 50% (8/16) were positive for aPL: four for criteria aPL, and four for non-criteria aPL. Criteria aPL positivity was significantly associated with an increased risk of VTE recurrence (OR 4.83, 95% CI 1.31-17.74).

Of note, the association between antiphospholipid antibody positivity and VTE recurrence should be interpreted with caution, as this analysis was based on a limited number of events (n = 16), resulting in wide confidence intervals. These findings are therefore considered exploratory and hypothesis-generating, and no definitive conclusions can be drawn regarding the magnitude or independence of this association.

Notably, 63% (10/16) of recurrent patients were not on anticoagulant therapy at the time of the event. Among the four patients with criteria aPL, two were on vitamin K antagonists, and two were not. Among the 35 non-criteria aPL positivity patients, 4 (11%) experienced recurrence, none of whom were anticoagulated.

Factors associated with thrombotic recurrences are detailed in **Supplementary Table 4**.

#### Chronic thromboembolic pulmonary hypertension

The analysis of chronic thromboembolic pulmonary hypertension (CTEPH) was performed exclusively among patients with objectively confirmed pulmonary embolism (PE), irrespective of whether concomitant deep vein thrombosis (DVT) was present. Patients with isolated DVT were excluded from this analysis, as DVT in the absence of PE is not associated with the development of CTEPH. The occurrence of CTEPH is determined by the pulmonary embolic event itself and is therefore independent of the coexistence of DVT.

Of the total VTE cohort, 27 patients presented with isolated DVT, 108 with isolated PE, and 55 with combined DVT and PE. The CTEPH analysis was conducted among the 163 patients with documented PE, comprising both those with isolated PE and those with PE associated with concomitant DVT, yielding a denominator of 163 patients at risk.

Ten out of 163 patients (%) developed chronic thromboembolic pulmonary hypertension (CTEPH), corresponding to an annual rate of %. The development of CTEPH was significantly associated with older age (median [82 IQR 79–86 vs. 74 years IQR 50-85 (p=0.047)]), respiratory failure during the acute phase of pulmonary embolism (OR 6.52, 95% CI 1.29-32.743), and positivity for IgA aB2GP1 (OR 5.7, 95% CI 1.53-21.40) (**Table 4**).

**Table 4:** Predictive factors associated with Chronic thromboembolic pulmonary hypertension (CTEPH): unadjusted OR.

Variable	CTEPH (n=10)	No CTEPH (n=153)	OR (95 CI%)	p
Age(years) Median (IQR)	82 (IQR 79-86)	74 (IQR 50-85)	**1.06 (1-1.12)**	**0.047**
Over 65 years	7 (70%)	85 (56%)	0.50 (0.12-2.03)	0.379
Gender (women)	8 (80%)	88 (58%)	3.04 (0.6-14.81	0.180
Hypertension	1 (10%)	24 (16%)	0.60 (0.73-5)	0.632
*Diabetes Mellitus*	3 (30%)	56 (37%)	0.73 (0.18-2.96)	0.675
Dyslipidemia	4 (40%)	60 (49%)	0.70 (0.188-2.6)	0.578
Obesity	1 (10%)	23 (15%)	0.60 (0.07-5.09)	0.666
Smoking	1 (10%)	16 (11%)	0.90 (0.1-7.56)	0.963
Alcohol	2 (20%)	22 (14%)	1.59 (0.31-8.03)	0.629
IBD	4 (40%)	44 (29%)	1.73 (0.46-6.45)	0.410
CVD	2 (20%)	13 (8.6%)	2.73 (0.52-14.22)	0.240
CKD	2 (20%)	49 (32%)	0.52 (0.1-2.56)	0.434
Neurologic disease	1 (10%)	12 (7.9%)	1.21 (0.14-10.34)	0.808
Cerebrovascular disease	1 (10%)	14 (9.2%)	0.96 (0.11-8.12)	0.928
COPD	2 (20%)	17 (11%)	2.02 (0.39-10.35)	0.404
Respiratory failure	7 (78%)	43 (34%)	**6.67 (1.32- 33.53)**	**0.021**
aCL IgG	0	6 (4%)	NE	
aCL IgM	0	8 (5.2%)	NE	
aB2GP1 IgG	0	4 (2.6%)	NE	
aB2GP1 IgM	0	9 (5.8%)	NE	
aB2GP1 IgA	5 (50%)	30 (20%)	**5.95 (1.59-22.27)**	**0.008**
aPS/PT IgG	0	7 (4.5%)	NE	
aPS/PT IgM	0	16 (11%)	NE	
LA	0	12 (8.3%)	NE	
Triple aPL positivity	0	3 (2%)	NE	

NE: Not evaluable, all controls are negative, much larger samples would be necessary to calculate it. Abbreviations: CKD, chronic Kidney disease; COPD, chronic obstructive pulmonary disease; aPL, antiphospholipid antibodies; aCL, anticardiolipin; aB2gp1, Anti-Beta2 glycoprotein 1; aPS/PT, anti-phosphatidylserine-prothrombin; LA, lupus anticoagulant. OR: Odds ratio, significant results are marked in bold.

#### Major bleeding

Major bleeding occurred in 5.5% of patients receiving anticoagulation therapy, corresponding to an annual rate of 2.7%. Gastrointestinal bleeding was the most frequent type of bleeding, accounting for 50% of all bleeding events. Additionally, there were two cases of intracranial bleeding (20%), one case of life-threatening epistaxis (10%), and two episodes of soft-tissue bleeding (20%).

The clinical factors significantly associated with the development of major bleeding included CKD (OR 5.17, 95% CI 1.16-22.98), anaemia (OR 6.59, 95% CI 1.57-27.66), and arterial hypotension during the acute phase of VTE (OR 4.46, 95% CI 1.01-19.72) (see **Supplementary Table 5**). Given the limited number of events, the associated estimates are characterised by wide confidence intervals reflecting substantial statistical imprecision. These findings should therefore be interpreted with caution and considered exploratory.

Both anaemia and arterial hypotension were recorded at baseline as part of the standard clinical assessment performed at the time of study inclusion, prior to the occurrence of any bleeding event.

## Discussion

To our knowledge, this is one of the first prospective studies specifically evaluating both criteria and non-criteria antiphospholipid antibodies (aPL) during the acute phase of venous thromboembolism (VTE), with blood samples obtained within the first 24 hours after diagnosis. Our findings suggest that, beyond antibodies included in the Sydney classification criteria (8), non-criteria antibodies—particularly IgA anti-β2-glycoprotein I (aβ2GPI)—may also be associated with clinical outcomes. These results align with emerging evidence supporting a potential role for non-criteria aPL within an expanded antiphospholipid syndrome (APS) spectrum (7, 20).

By distinguishing between provoked and unprovoked VTE, in accordance with contemporary classification approaches (9), our study reinforces the established role of criteria aPL while also identifying an independent association between IgA aβ2GPI positivity and unprovoked VTE, as well as disease severity. Antiphospholipid antibody positivity is a well-recognized risk factor for VTE (10), associated not only with the development of a first thrombotic event (33, 34) but also with recurrence (32, 33). Consistent with prior studies, criteria aPL were significantly associated with first VTE in our cohort.

Although the prevalence of aPL, including IgA aβ2GPI, may increase with age (24), patients with VTE exhibited higher aPL prevalence than age-adjusted controls. While this may partially reflect shared vascular risk factors such as hypertension and dyslipidemia (27, 28), multivariate analysis confirmed that IgA aβ2GPI positivity was independently associated with unprovoked VTE. Nevertheless, this association should be interpreted with caution, as prior studies have yielded inconsistent results, likely due to heterogeneity in study design, patient populations, and laboratory methodologies (19, 20).

IgA aβ2GPI positivity was also associated with a more severe clinical presentation, as reflected by its relationship with higher-risk pulmonary embolism severity index (PESI) categories, a validated prognostic tool in VTE (29). Although this finding suggests a potential role for IgA aβ2GPI in modulating disease severity, the underlying mechanisms remain uncertain and require further investigation.

The slightly younger age of the control group reflects the difficulty of recruiting healthy individuals over 85 years; however, the proportion of subjects above and below 65 years was comparable, and this age imbalance did not significantly affect the identification of independent predictors.

Mortality in our cohort was associated with advanced age, severe initial presentation, and comorbidities, consistent with previous reports in VTE populations (30, 31).

Baseline anaemia was identified as a predictor of major bleeding in our cohort, consistent with its established role as an independent risk factor in validated VTE-specific bleeding scores, including the RIETE (43) VTE-BLEED (44) and HEMORR_2_HAGES (45) scores. Regarding arterial hypotension, to the best of our knowledge no published bleeding risk score specifically developed for VTE populations has incorporated this variable as an independent predictor of major bleeding. The observed association in our cohort may therefore not reflect a direct causal relationship, but rather an indirect marker of underlying disease severity — such as haemodynamically significant pulmonary embolism — or of substantial comorbidity burden, both of which may independently contribute to an increased bleeding risk. This hypothesis-generating finding warrants further investigation in larger prospective studies with detailed haemodynamic characterisation.

Criteria aPL positivity and triple aPL positivity were associated with an increased risk of recurrence, in line with previous studies (32, 33). Development of chronic thromboembolic pulmonary hypertension (CTEPH) was associated with age and respiratory failure during the acute event, consistent with existing literature (35, 36). Although criteria aPL have been previously linked to CTEPH (37–39), we did not observe a significant association, likely due to limited statistical power. In contrast, IgA aβ2GPI showed an association with CTEPH, although this finding should be interpreted cautiously given the small number of events.

Chronic kidney disease and anaemia were associated with major bleeding, consistent with established definitions and risk factors in anticoagulated patients (40, 41). As previously reported, no clear association was observed between bleeding risk and aPL profile (32).

This study has several limitations that should be considered when interpreting the results. First, the sample size may have limited the statistical power to detect independent associations for less frequent outcomes, such as thrombotic recurrence, chronic thromboembolic pulmonary hypertension (CTEPH), or major bleeding. Consequently, some observed associations—particularly those involving IgA anti-β2GPI—should be interpreted with caution.

Second, this was a single-centre cohort including only hospitalized patients with acute VTE, which may limit the generalizability of our findings to patients with less severe disease managed in outpatient settings.

Third, the exclusion of patients with active malignancy represents a limitation of this study, restricting the generalisability of our findings to the cancer-associated VTE population. Given the distinct thrombotic pathophysiology of cancer-associated thrombosis and the recognised, though incompletely understood, prevalence of antiphospholipid antibody positivity in neoplastic patients, the applicability of our findings to this subgroup cannot be assumed and warrants dedicated investigation in future studies.

Forth, lupus anticoagulant (LA) testing was not performed in all patients. This was due to the well-recognized limitations of LA determination during the acute phase of VTE, where results may be affected by anticoagulant therapy, acute-phase responses, and assay interference, potentially leading to false-positive or false-negative results (8, 13).

Fifth, not all patients with positive antiphospholipid antibodies underwent confirmatory testing after 12 weeks, mainly due to logistical constraints during the COVID-19 pandemic. This may have led to overestimation of persistent antiphospholipid antibody positivity. However, among retested individuals, antibody persistence was confirmed in the large majority of cases consistent with previous reports (34, 42). Variability in a minority of cases may reflect values near assay cut-offs or analytical variability rather than true negativization.

Sixth, although multivariate models were used to adjust for known confounders, residual confounding cannot be excluded. In particular, unmeasured prothrombotic factors or inflammatory states may have influenced the observed associations between IgA anti-β2GPI and VTE.

A further limitation of this study is the small number of recurrent VTE events available for analysis (n = 16), which substantially reduces the statistical power of the recurrence-related estimates and results in wide confidence intervals. Consequently, the observed association between antiphospholipid antibody positivity and VTE recurrence must be interpreted with caution. Larger prospective cohort studies are needed to confirm these findings and provide more precise estimates of the true effect size.

Finally, the observational design of the study precludes establishing causality. Therefore, the association between IgA anti-β2GPI and thrombotic events should be interpreted as hypothesis-generating rather than confirmatory.

Despite these limitations, our study provides relevant insights. By focusing on a non-oncologic, non-autoimmune VTE population, we demonstrate that APS-related serological profiles may be more prevalent than traditionally assumed, affecting a substantial proportion of patients irrespective of age (22). In addition, IgA aβ2GPI antibodies were the most prevalent aPL during the acute phase of VTE, consistent with findings in other thrombotic conditions such as ischemic stroke (21).

From a pathophysiological perspective, our findings may be interpreted within an expanded mechanistic framework of APS. Beyond the criteria mechanisms attributed to aPL—including endothelial and platelet activation, complement engagement, and neutrophil extracellular trap formation—β2-glycoprotein I (β2GPI) plays a central role as the main antigenic target and a modulator of coagulation and immune responses (12, 14–17, 23). Although antigen recognition is primarily determined by antibody specificity, isotype-related differences in effector function may significantly influence pathogenic potential. While IgA and IgG anti-β2GPI antibodies may recognize similar epitopes, IgA antibodies interact with distinct Fc receptors and exhibit limited ability to activate the criteria complement pathway (18), suggesting that IgA aβ2GPI antibodies may promote thrombosis through complement-independent mechanisms.

Within this context, IgA aβ2GPI antibodies may contribute to thrombosis through immune complex formation and functional interference with β2GPI. Such interactions could reduce the bioavailability or alter the conformation of β2GPI, thereby impairing its anticoagulant and immunomodulatory functions (16, 17). This hypothesis is biologically plausible and supported by indirect evidence linking reduced β2GPI levels or dysfunction to prothrombotic states in inflammatory conditions (25, 26), although whether these alterations represent a causal mechanism or a secondary epiphenomenon remains uncertain.

These mechanistic considerations are consistent with our clinical findings, which demonstrate an independent association between IgA aβ2GPI antibodies and VTE, including unprovoked events. However, causality cannot be established, and residual confounding or unmeasured prothrombotic factors may contribute to this association. Moreover, prior studies have reported heterogeneous results regarding the clinical significance of IgA aPL (19, 20).

Importantly, IgA anti-β2GPI antibodies remain classified as non-criteria aPL and are not included in current APS classification criteria (8, 9), reflecting ongoing concerns regarding assay variability, lack of standardization, and conflicting evidence supporting their pathogenic role (20). In addition, aPL—including IgA isotypes—may be detected in healthy individuals and transient inflammatory states (24), raising the possibility that, in some contexts, they may represent epiphenomena rather than direct mediators of thrombosis.

Finally, the differential role of IgA aβ2GPI in arterial versus venous thrombosis deserves consideration. While previous studies have more consistently associated these antibodies with arterial events, our findings suggest a potential role in venous thrombosis. This raises the possibility that distinct mechanisms may predominate depending on the vascular bed, with immunothrombotic pathways playing a more prominent role in venous disease (23). However, this interpretation remains speculative and requires validation in mechanistic and longitudinal studies.

Further studies with larger cohorts are needed to validate these findings, clarify the role of aPL in recurrence, and determine their contribution to VTE-related complications such as CTEPH.

Finally, our results raise an important clinical question: should aPL testing remain restricted to younger patients with VTE? Given the potential prognostic implications of aPL positivity—and the observed association of IgA aβ2GPI with unprovoked VTE, disease severity, and CTEPH—future studies should evaluate whether broader testing strategies could improve risk stratification and clinical management.

## Conclusion

Our study shows that IgA anti-β2-glycoprotein I (IgA aβ2GPI) antibodies are frequently detected during the acute phase of venous thromboembolism (VTE) and are independently associated with unprovoked events and greater clinical severity. In addition, an association with chronic thromboembolic pulmonary hypertension (CTEPH) was observed, although this finding should be interpreted with caution.

These results support a potential role for IgA aβ2GPI within the broader spectrum of antiphospholipid antibodies in VTE. However, given the limitations of the study, their clinical and prognostic utility remains to be established.

Further prospective studies are needed to confirm these findings and to clarify the role of IgA aβ2GPI in risk stratification and clinical management.

## Data Availability

The original contributions presented in the study are included in the article/**Supplementary Material**. Further inquiries can be directed to the corresponding author/s.
