# Supplementary material for: IgA anti-β2-glycoprotein I as an independent risk factor in acute venous thromboembolism
Source: Front Immunol. 2026 Jul 20;17:1818399. doi: 10.3389/fimmu.2026.1818399 (PMC13429589; doi:10.3389/fimmu.2026.1818399)
Supplement: Supplementary file 1 [file SupplementaryFile1.docx]

Supplementary Material

# Supplementary Tables

Supplementary Table S1. Confirmation of aPL positivity. Of the 51 positives in the first determination, a second sample was evaluated in 35 (69%).

Supplementary Table S2. Comorbidities and aPL distribution in patients with unprovoked VTE compared to controls.

Supplementary Table S3. Mortality in VTE patients. Characteristics of deceased patients versus survivors. Predictive factors associated with exitus: unadjusted OR.

Supplementary Table S4 Characteristics of patients with VTE recurrence: unadjusted OR.

Supplementary Table S1. Confirmation of aPL positivity. Of the 51 positives in the first determination, a second sample was evaluated in 35 (69%).

| **Antibodies** | **Positives in first determination** | **Positives in second determination** | **Confirmation of positivity** |
| --- | --- | --- | --- |
| IgG aCL | 5 | 4 | 80% |
| IgM aCL | 3 | 3 | 100% |
| IgG aB2gp1 | 3 | 3 | 100% |
| IgM aB2gp1 | 5 | 5 | 100% |
| IgA aB2gp1 | 19 | 18 | 95% |
| IgG APS/PT | 6 | 5 | 83% |
| IgM APS/PT | 11 | 8 | 73% |
| Any Classic aPL | 8 | 8 | 100% |
| Any Extra criteria aPL | 31 | 27 | 87% |
| Any APL positive | 35 | 31 | 89% |
| Lupus anticoagulant | 9 | 8 | 89% |

The degree of confirmation in each antibody is indicate. Abbreviations: aPL, antiphospholipid

antibodies; aCL, anticardiolipin; aB2gp1, Anti-Beta2 glycoprotein 1.

aPS/PT, anti-phosphatidylserine-prothrombin.

Supplementary Table S2. Comorbidities and aPL distribution in patients with unprovoked VTE compared to controls.

| **Parameter** | **Unprovoked VTE Patients**  **(n=114)** | **Controls**  **(n=181)** | **OR (CI 95%)** | **p(value)** |
| --- | --- | --- | --- | --- |
| Age over 65 years | 79 (69%) | 108 (60%) | 1.53 (0.93-2.51) | 0.095 |
| Gender (women) | 47 (41%) | 78 (43%) | 0.92 (0.57-1.49) | 0.752 |
| Hypertension | 68 (60%) | 76 (37%) | **2.51 (1.55-4.06)** | **<0.001** |
| Diabetes | 20(16%) | 27(15%) | 1.21 (0.64-2.28) | 0.549 |
| Dyslipidemia | 42 (37%) | 47 (26%) | **1.66 (1.00-2.75)** | **0.048** |
| Smoking | 38 (33%) | 25 (14%) | **3.12 (1.75-5.54)** | **<0.001** |
| Obesity | 47 (52%) | 20(11%) | **8.59 (4.62-15.99)** | **<0.001** |
| Classic aPL (Sydney criteria) | 11 (9.6%) | 4 (2.2%) | **4.72 (1.46-15.22)** | **0.009** |
| IgG aCL | 5 (4.4%) | 0 (0%) | NE |  |
| IgM aCL | 4 (3.5%) | 4 (2.2%) | 1.60 (0.39-6.56) | 0.507 |
| IgG aB2GP1 | 4 (3.5%) | 0 (0%) | NE |  |
| IgM aB2GP1 | 6 (5.3%) | 3 (1.7%) | 3.29 (0.80-13.45) | 0.096 |
| IgA aB2GP1 | 23 (20%) | 9 (5%) | **4.83 (2.14-10.83)** | **<0.001** |
| aPS/PT (IgG or IgM) | 12(10.5%) | 12 (6.6%) | 1.65 (0.71-3.82) | 0.237 |
| IgG aPS/PT | 5 (4.4%) | 5 (2.8%) | 1.61 (0.45-5.70) | 0.475 |
| IgM aPS/PT | 9 (7.9%) | 7 (3.9%) | 3.13 (0.77-5.89) | 0.145 |
| Any aFL | 34 (30%) | 24(13%) | **2.78 (1.54-5.00)** | **0.001** |
| Lupus anticoagulant | 7 (7%) | 10 (6.3%) | 1.20 (0.44-3.24) | 0.724 |
| Triple aPL positive | 3 (2.6%) | 0 (0%) | NE |  |

NE: Not evaluable, all controls are negative, much larger samples would be necessary to calculate it.

Abbreviations: aPL, antiphospholipid antibodies; aCL, anticardiolipin; aB2gp1, Anti-Beta2 glycoprotein 1; aPS/PT, anti-phosphatidylserine-prothrombin. NE not evaluable. OR: Odds ratio, significant results are marked in bold.

Supplementary Table S3. Mortality in VTE patients. Characteristics of deceased patients versus survivors. Predictive factors associated with exitus: unadjusted OR.

| **Variable** | **Exitus (n=34)** | **No Exitus (n=147)** | **OR (IC 95%)** | **p** |
| --- | --- | --- | --- | --- |
| Age (years) Median (IQR) | 86 (IQR 76-90) | 72 (IQR 49-83) | **1.05 (1.02-1.09)** | **<0.001** |
| Over 65 years | 32 (94%) | 109 (74%) | **3.96 (1.49-10.48)** | **0.006** |
| Gender (%women) | 22 (65%) | 79 (54%) | 0.62 (0.28-1.35) | 0.248 |
| Hypertension | 26 (77%) | 79 (54%) | 2.79 (1.18-6.58) | **0.019** |
| *Diabetes Mellitus* | 7 (21%) | 20 (14%) | 1.63 (0.62-4.24) | 0.307 |
| Dyslipidemia | 13 (38%) | 52 (35%) | 1.11 (0.51-2.41) | 0.754 |
| Obesity | 18 (58%) | 52 (46%) | 1.65 (0.74-3.70 | 0.237 |
| Smoking | 4 (12%) | 22 (15%) | 0.75 (0.24-2.34) | 0.623 |
| Alcohol | 2 (5.9%) | 17 (12%) | 0.47 (0.1-2.15) | 0.340 |
| IBD | 1 (2.9%) | 3 (2.1%) | 1.44 (0.14-14.33) | 0.749 |
| CVD | 14 (41%) | 39 (272%) | 1.9 (0.88-4.16) | 0.094 |
| CKD | 4 (12%) | 13 (9%) | 1.36 (0.41-4.47) | 0.600 |
| Neurologic disease | 20 (59%) | 34 (23%) | **4.74 (2.16-10.39)** | **<0.001** |
| COPD | 5 (15%) | 14 (9.5%) | 1.60 (0.54-4.87) | 0.378 |
| OSA | 1 (2.9%) | 19 (13%) | 0.20 (0.02-1.56) | 0.128 |
| Previous VTE | 3 (8.8%) | 23 (16%) | 0.76 (0.16-3.63) | 0.308 |
| Anemia | 9 (27%) | 39 (27%) | 0.98 (0.42-2.3) | 0.994 |
| Bleeding | 3 (10%) | 7 (5.3%) | 2 (0.48-8.23) | 0.330 |
| Respiratory failure | 18 (70%) | 39 (36%) | **4.67 (1.86-11.68)** | **0.001** |
| aB2GP1 IgG | 1 (2.9%) | 4 (2.7%) | 1.07 (0.11-9.94) | 0.944 |
| aB2GP1 IgM | 2 (5.9%) | 7 (4.8%) | 1.24 (0.24-6.25) | 0.787 |
| aB2GP1 IgA | 9 (27%) | 20 (14%) | 2.28 (0.93-5.60) | 0.071 |
| aCL IgG | 1 (2.9%) | 6 (4.1%) | 0.7 (0.08-6.07) | 0.757 |
| aCL IgM | 1 (2.9%) | 7 (4.8%) | 0.6 (0.07-5.06) | 0.645 |
| aPS/PT IgG | 3 (8.8%) | 6 (4.1%) | 2.25 (0.53-9.52) | 0.263 |
| aPS/PT IgM | 5 (15%) | 11 (7.5%) | 2.11(0.68-6.55) | 0.189 |
| Lupus anticoagulant | 2 (6.5%) | 11 (8.1%) | 0.78 (0.16-3.72) | 0.767 |

Abbreviations: IBD, intestinal bowel disease; CVD, cardiovascular disease; CKD, chronic kidney disease; COPD chronic obstructive pulmonary disease; OSA, obstructive sleep apnea; aCL, anticardiolipin; aB2gp1, Anti-Beta2 glycoprotein 1; aPS/PT, anti-phosphatidylserine-prothrombin. OR: Odds ratio, significant results are marked in bold.

Supplementary Table S4 Characteristics of patients with VTE recurrence: unadjusted OR.

| **Variable** | **Recurrence**  **(n=16)** | **No recurrence**  **(n=155)** | **OR (95 CI%)** | **p** |
| --- | --- | --- | --- | --- |
| Age(years) Median (IQR) | 72 (IQR 39-86) | 76 (IQR 55-85) | 0.98(0.96-1.00) | 0.230 |
| Over 65 years | 10 (63%) | 122 (79%) | 0.70 (0.25-2.03) | 0.530 |
| Gender (%women) | 9 (56%) | 88 (43%) | 1.03 (0.36-2.91) | 0.995 |
| Hypertension | 8 (50%) | 92 (59%) | 0.60 (0.24-1.89) | 0.472 |
| *Diabetes Mellitus* | 2(13%) | 23 (15%) | 0.82 (0.17-3.87) | 0.801 |
| Dyslipidemia | 4 (25%) | 59 (38%) | 0.53 (0.16-1.73) | 0.308 |
| Obesity | 4 (25%) | 65 (53%) | 0.30 (0.09-1.07) | 0.069 |
| Smoking | 5 (31%) | 20 (13%) | 3.13 (0.98-9.92) | 0.053 |
| Alcohol | 4 (25%) | 14 (9%) | 3.42 (0.97-12.05) | 0.059 |
| IBD | 3 (19%) | 21(14%) | 1.48 (0.39-5.64) | 0.571 |
| CVD | 2 (13%) | 48(31%) | 0.31 (0.06-1.42) | 0.130 |
| CRD | 1 (6%) | 16 (10%) | 0.58 (0.07-4.71) | 0.608 |
| Neurologic disease | 4 (25%) | 46 (30%) | 0.77 (0.23-2.52) | 0.696 |
| Cerebrovascular disease | 1 (6%) | 13 (8%) | 0.80 (0.09-6.58) | 0.831 |
| COPD | 2 (13%) | 15 (10%) | 1.34 (0.27-6.48) | 0.652 |
| OSA | 2 (12%) | 42 (27%) | 0.38 (0.08-1.77) | 0.384 |
| Previous VTE | 3 (19%) | 17 (11%) | 1.88 (0.48-7.29) | 0.350 |
| Anemia | 1 (6%) | 23 (15%) | 0.38 (0.04-3.03) | 0.360 |
| Bleeding | 6 (43%) | 83 (66%) | 0.37 (0.12-1.15) | 0.090 |
| HBPM | 1 (6%) | 16 (10.3%) | 0.54 (0.06-4.38) | 0.608 |
| Acenocoumarol | 9 (56%) | 87 (56%) | 1.02 (0.36-2.87) | 0.993 |
| Rivaroxaban | 7 (44%) | 38 (25 %) | 2.41 (0.84-6.92) | 0.104 |
| Apixaban | 1 (6%) | 16 (10%) | 0.50 (0.07-4.71) | 0.608 |
| aCL IgG | 2 (13%) | 4 (3%) | 5.42 (0.91-32.30) | 0.060 |
| aCL IgM | 2 (13%) | 5 (3%) | 4.31 (0.76-24.30) | 0.090 |
| aB2GP1 IgG | 1 (6%) | 3 (1.9%) | 3.40 (0.33-34.75) | 0.305 |
| aB2GP1 IgM | 2 (13%) | 6 (4%) | 3.57(0.65-19.38) | 0.142 |
| Classical aPL | 4 (25%) | 10(7%) | **4.83 (1.31-17.74)** | **0.018** |
| aB2GP1 IgA | 3 (19%) | 24 (16%) | 1.20 (0.32-4.55) | 0.734 |
| aPS/PT IgG | 2 (13%) | 6 (4%) | 3.57(0.65-19.38) | 0.140 |
| aPS/PT IgM | 3 (19%) | 13 (8%) | 2.53 (0.64-10.06) | 0.180 |
| Lupus anticoagulant | 2 (13%) | 10 (7%) | 2.07(0.41-10.5) | 0.370 |

Abbreviations: IBD, intestinal bowel disease; CVD, cardiovascular disease; CKD, chronic kidney disease; COPD chronic obstructive pulmonary disease; OSA, obstructive sleep apnea; aPL, antiphospholipid antibodies; aCL, anticardiolipin; aB2gp1, Anti-Beta2 glycoprotein 1; aPS/PT, anti-phosphatidylserine-prothrombin. OR: Odds ratio, significant results are marked in bold.
