# Supplementary material for: IgA anti-β2-glycoprotein I as an independent risk factor in acute venous thromboembolism
Source: Front Immunol. 2026 Jul 20;17:1818399. doi: 10.3389/fimmu.2026.1818399 (PMC13429589; doi:10.3389/fimmu.2026.1818399)

Supplementary Material

# Supplementary Figures

Figure S1. Algorithm and distribution of the patients.


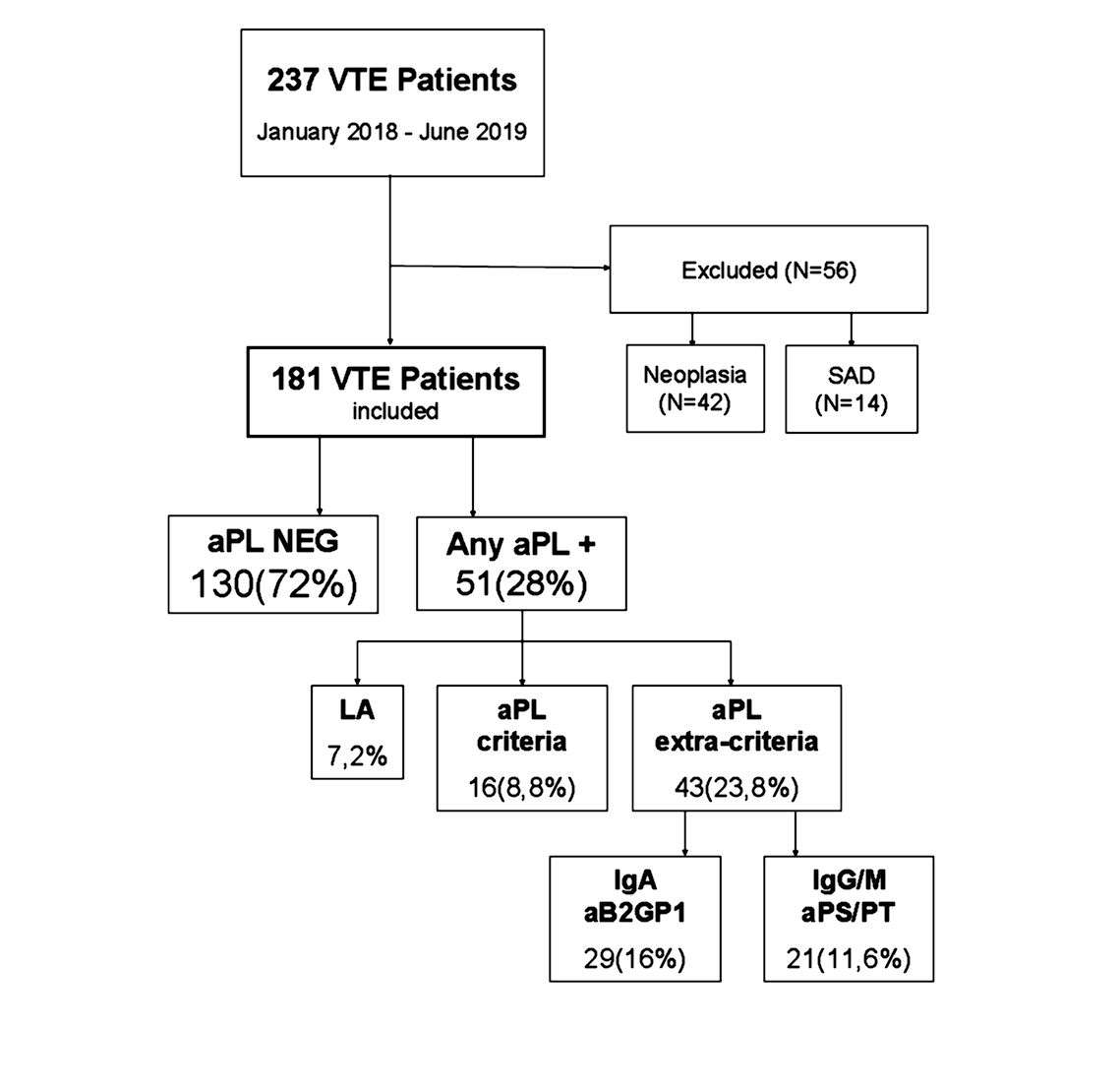


Figure S2. Distribution of aPL in controls and patients. The cutoff points for each aPL are marked with a red line


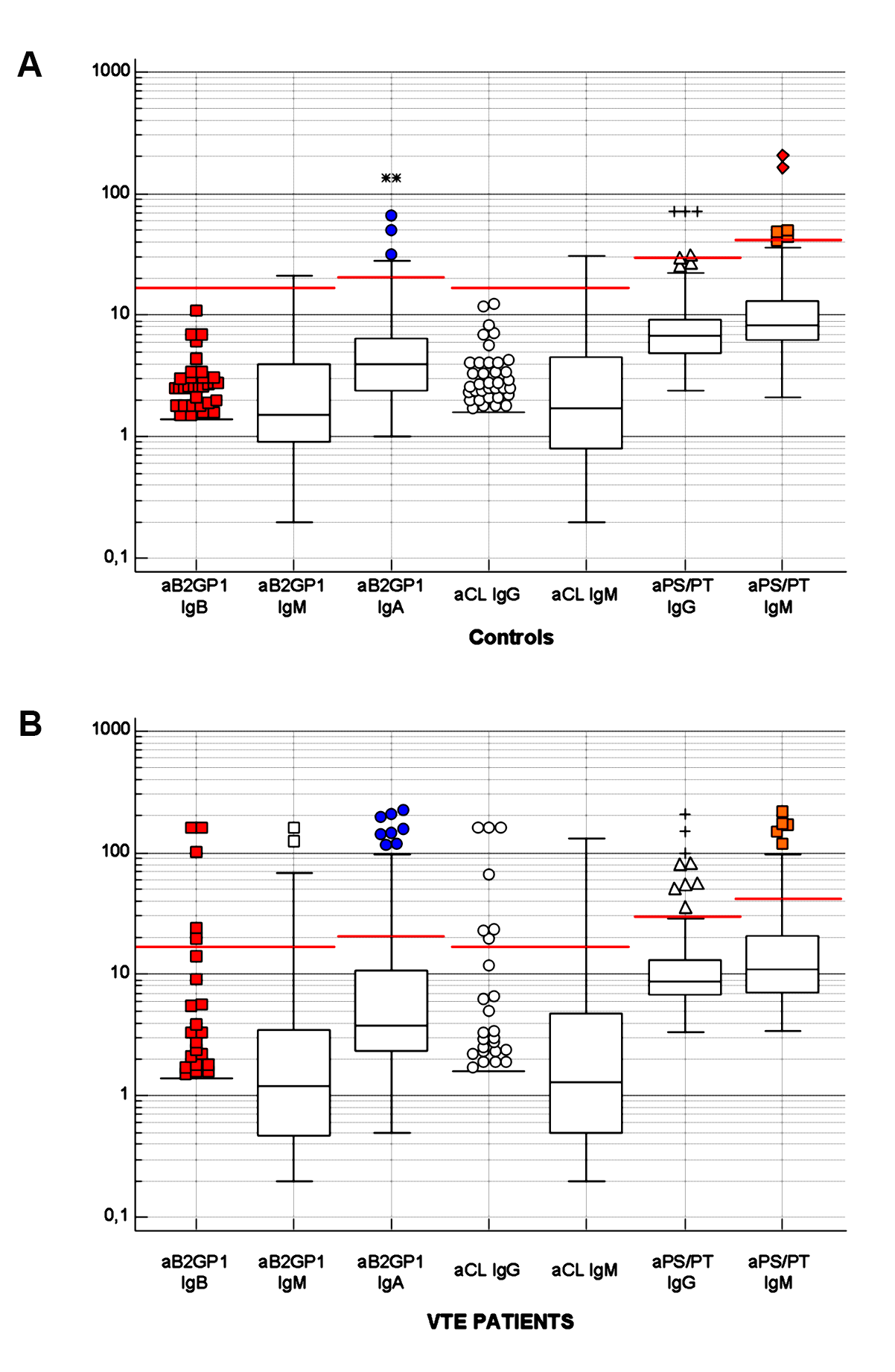

Supplement: Supplementary file 2 [file SupplementaryFile2.docx]
